# Muscular Anatomy of the *Podocoryna carnea* Hydrorhiza

**DOI:** 10.1371/journal.pone.0072221

**Published:** 2013-08-14

**Authors:** Leo W. Buss, Christopher Anderson, Edward W. Bolton

**Affiliations:** 1 Department of Ecology and Evolutionary Biology, Yale University, New Haven, Connecticut, United States of America; 2 Department of Geology and Geophysics, Yale University, New Haven, Connecticut, United States of America; UC Irvine, United States of America

## Abstract

The muscular anatomy of the athecate hydroid *Podocoryna carnea* hydrorhiza is elucidated. The polyp-stolon junction is characterized by an opening, here called the chloe, in the otherwise continuous hydrorhizal perisarc. The chloe is elliptical when the polyp first arises, but takes on a more complex outline as multiple stolons anastomose to communicate with that polyp. Surrounding the polyp base are spots, here called anchors, which autofluoresce at the same wavelengths as perisarc and which, like perisarc, contain chitin as assessed by Calcofluor White, Congo Red and wheat germ agglutinin staining. Anchors remain after living tissues are digested using KOH. Collagen IV staining indicates that the mesoglea is pegged to the anchors and rhodamine phallodin staining detects cytoskeletal F-actin fibers of the basal epidermis surrounding the anchors. Longitudinal muscle fibers of the polyp broaden at the polyp base and are inserted into the mesoglea of the underlying stolon, but were neither observed to extend along the stolonal axis nor to attach to the anchors. Circular muscular fibers of the polyp extend into stolons as a dense collection of strands running along the proximal-distal axis of the stolon. These gastrodermal axial muscular fibers extend to the stolon tip. Epidermal cells at the stolon tip and the polyp bud display a regular apical latticework of F-actin staining. A similar meshwork of F-actin staining was found in the extreme basal epidermis of all stolons. Immunohistochemical staining for tubulin revealed nerves at stolon tips, but at no other hydrorhizal locations. These studies bear on the mechanisms by which the stolon tip and polyp bud pulsate, the manner in which the stolon lumen closes, and on the developmental origin of the basal epidermis of the hydrorhiza.

## Introduction

Hydrozoans typically adhere to surfaces by hydrorhizal attachment structures. These may take the form of a basal disc, a system of root-like rhizoids, or an extensive system of stolons. Stolons are diploblastic tubes suspended from an encasing acellular perisarc and bound to the surface by basal epidermal rivets [1]. The stolonal lumen is co-extensive with that of the gastrovascular cavities of the polyps of the colony. In many encrusting forms the stolons elongate, branch, and anastomose to form a complex gastrovascular network. *Podocoryna carnea*, the object of this investigation, is such a hydroid.

Hydrorhizal tissues display coordinated patterns of movement. Fluids within the gastrovascular hydrorhizal network are driven by contractions of the polyps [2]–[5]. The radius of the stolonal lumen undergoes cycles of expansion and contraction, as fluids enter from the polyp and return to it. In addition to the bi-directional gastrovascular flows, the stolon tip and the polyp bud display growth pulsations [6]–[10]. The tip undergoes rhythmic changes in shape as it progresses over the surface [11], [12], as does the bud as it develops from a rudiment to a polyp [12]–[14]. Severed stolons continue to elongate and isolated buds continue to develop so are driven by motility systems distinct from the polyp contractions that drive gastrovascular flows [8], [9], [15], [16].

Curiously, the physiological and anatomical basis for these movement patterns have been little investigated. We have sought to ask whether the polyp-stolon junction is morphologically specialized, whether the cells of the stolons bear muscle fibers and, if so, how they are related to muscle fibers of polyp epitheliomuscular cells, and finally, whether the stolons bear specializations at the growing tip and polyp bud. Our principal approach has been the use of confocal microscopy with phalloidin-linked fluorescent dyes labeling F-actin to visualize muscle fibers and cytoskeletal features, supplemented by immunohistochemical stains to visualize mesoglea and nerves. We find the polyp-stolon junction to be anatomically simple, the stolon to bear extensive muscular fibers co-extensive with the circular muscle fibers of the polyps and the apical regions of tip and bud epidermal cells to support a distinctive F-actin meshwork reminiscent of adherens junction rings.

## Methods

Animal care at Yale University is conducted under the supervision of the Yale Animal Care and Use Committee. Hydroids are diploblastic organisms lacking a brain and are not governed by specialized guidelines. Animals sacrificed for confocal microscopy were clonal explants of animals maintained in continuous culture and were narcotized prior to fixation as detailed below.

Colonies of *Podocoryna carnea* (P3 strain) were maintained under standard conditions [17]. Briefly, colonies are grown on glass microscope slides, glass cover slips, or glass-bottomed Petri dishes. Clonal replicates are generated by explanting a small region of the hydrorhiza bearing 1–3 polyps and affixing them to a glass surface with a loop of quilting thread. After 2 days the colonies have attached and the thread is removed. Colonies are maintained in recirculating aquaria with daily exchanges of 25% of the seawater (31 ppm). Colonies are fed to repletion every other day with 3–4 day old *Artemia salina* nauplii.

To prepare specimens for microscopy, colonies growing on cover slips were relaxed in 2% urethane (Sigma) for 90 seconds, then fixed in 4% formaldehyde for 2 hours at room temperature. After fixation, colonies were washed 3×5 minutes in 1× PBS, and permeabilized in 0.5% Triton in 1× PBS for 10 minutes at room temperature. Colonies were washed an additional 3×5 min in 1× PBS, after which they were blocked with 3% dry milk in 1× PBS for 1 hour at 4 degrees C. To stain for F-actin, colonies were washed once in 1× PBS and stained with a 1∶40 dilution of rhodamine phalloidin (Biotium #00027, Hayward CA) for 30 minutes at room temperature. After 2×5 min washes in 1× PBS the coverslips bearing colonies were mounted inverted on microscope slides in ProLong Gold (Invitrogen, Eugene OR), supported on two thin strips of double stick tape.

To detect mesoglea, we stained for collagen IV using a mouse monoclonal antibody (M-39) kindly provided by Xiaoming Zhang [18], [19]. Nerves were visualized by detecting tubulin using a 1∶1 mixture of mouse monoclonal supernatants 12 g10 and E7 (Developmental Studies Hybridoma Bank, University of Iowa, Iowa City, IA). Colonies were treated as with rhodamine phalloidin staining up to and including the permeabilization step. Thereafter colonies were blocked with 10% goat serum in 1× PBS for 1 hour at room temperature. Blocking solution was replaced with type IV collagen antibody at 1∶400 in 10% goat serum/1×PBS or the tubulin antibody mixture at 1∶10 and incubated overnight at 4 degrees C. The colonies were then washed 3×5 minutes in 1× PBS, and incubated with secondary antibody (1∶80 FITC goat anti mouse, Invitrogen) with or without rhodamine phalloidin (1∶100, Biotium) in a 10% goat serum/1× PBS solution for 2 hours at room temperature. After 2×5 min washes in 1× PBS the coverslips were mounted as described above.

In some preparations nuclei were stained prior to mounting using Hoechst 33342 (Molecular Probes, 1∶200) or DAPI. Cover slips were immersed in Hoechst stain for 20 minutes followed by 2×5 min 1× PBS washes. For DAPI staining, a ProLong Gold product containing DAPI was used.

Unless otherwise noted images were captured on a Zeiss LSM 500 laser scanning confocal microscope. Confocal images were acquired and processed using either Zeiss Axiovision or Zeiss Efficient Navigation (ZEN) software. Projection and three-dimensional rendering were performed using this same software. Optical sections were made through 152 polyp-stolon junctions, 149 stolons, and 18 polyp buds.

Perisarc was studied in animals from which tissues were removed by KOH digestion. To remove tissue, colonies were relaxed for 90 sec. in 2% urethane (Sigma) and placed in dH_2_O for 10 minutes in a Coplin jar. Colonies are then placed horizontally in petri dishes and digested for 10–15 minutes in 10% KOH with intermittent gentle rinsing of surface with the KOH solution using a Pastuer pipette until tissue has digestion was complete (ca. 10–15 min). Slides were then washed 3×2 minutes in dH_2_O. Perisarc autofluorescence was documented at blue (Ex., 760–780 in 2-photon configuration, chameleon IR laser, no pinhole, Em. 435–485,), green (Ex., 488 nm, Em. 500–550 nm), and red (Ex., 561 nm, Em. 575 nm) wavelengths.

Perisarc contains chitin [20], [21]. Three conventional chitin stains were employed for chitin staining. Calcofluor White binds to chitin, cellulose and other β-1,4-linked carbohydrates [22], [23]. To stain using Calcofluor White, KOH treated slides were placed, inverted, over ca. 1 ml of 1 g/l solution of Calcofluor White solution (Fluka Analytical, Sigma) in a glass-bottomed petri dish (Warner Instruments). Following a one minute incubation, stolons were immediately imaged using a DAPI filter set (Ex 365 nm, Em 450 nm) on a Zeiss Axiovert microscope. Congo Red stains chitin and collagen [24]–[26]. Perisarc does not contain collagen [27]. KOH treated colonies were stained overnight at room temperature in a Coplin jar containing a filtered 1.5 mg/ml solution of Congo Red (Sigma) in dH_2_O. Coverslips were rinsed until no further staining solution could be seen in the wash, and imaged in dH_2_O on a Zeiss LSM 500 confocal microscope. Finally, wheat germ agglutinin binds to the N-acetyl-D-glucosamine residues of chitin. To stain with wheat germ agglutinin, slides were incubated for 20 minutes at room temperature in 25 µg/ml WGA-AlexaFluor 555 (Invitrogen) in PBS, washed 3×5 minutes in PBS, imaged in dH_2_O using a Texas Red filter set (Ex 560 nm, Em 645 nm) on a Zeiss LSM 500 confocal microscope.

We found it useful to image hyperplastic stolons. These are specialized stolonal tips that are differentiated in either xenogeneic encounters with *Hydractinia symbiolongicarpus* or allogeneic encounters with unrelated *P. carnea* colonies [28]–[30]. The tip region is extended and the encasing periderm reduced in hyperplastic stolons [29], offering the opportunity to image tip-specific features more fully. To study hyperplastic stolons, we established clonal explants of *P. carnea* and *H. symbiolongicarpus* on a common surface, which induced development of hyperplastic stolons for imaging.

## Results

### The Polyp-Stolon Junction

#### The chloe

**Figure 1:**
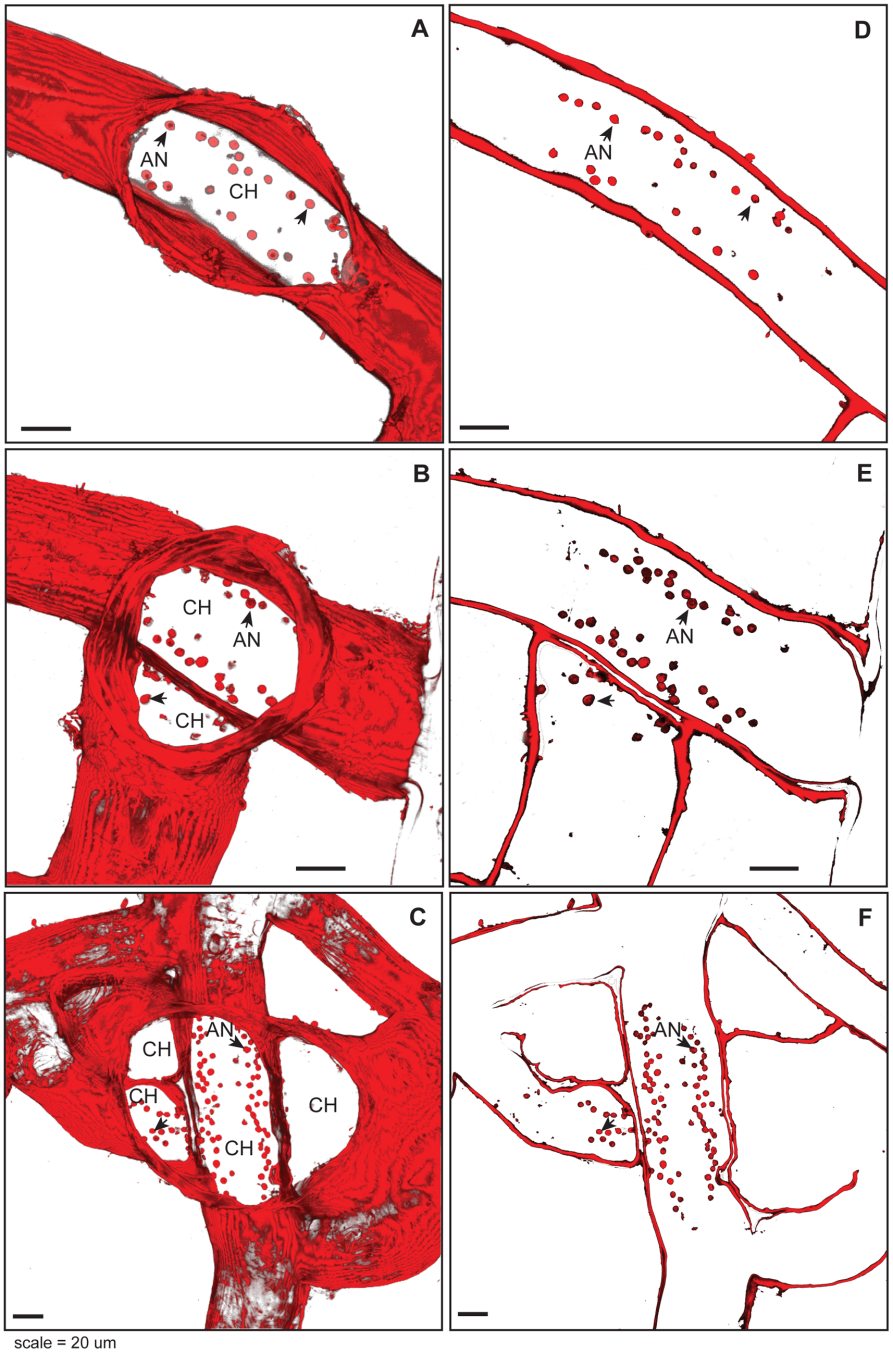
Chloe and anchors. Perisarc stained with WGA, tissues KOH digested. (A–C) Projections of chloe from polyps of differing age and connectivity with surrounding hydrorhiza. Optical depth, number of sections: (A) 32 µm, 17; (B) 34 µm, 18; (C) 38 µm, 20. (D–F) Optical section of same polyp-stolon junctions shown in (A–C) at ca. 3 µm above the substratum, showing anchors (arrowheads). (A–F) AN, anchors; CH, Chloe, Scale: 20 µm.

Polyps emerge from a hole in the otherwise continuous perisarc. This opening appears to lack a name and, since we will have occasion to refer to it frequently, we here designate this opening as the chloe. In young polyps the chloe was found to be elliptical in outline (Fig. 1A), with the major axis aligned with the proximal-distal axis of the stolon. In older polyps, where stolon branches have grown into locations near a polyp's base, the chloe appeared as an opening communicating with both the original polyp and lumen(s) of adjacent stolons (Fig 1B,C).

#### Anchors

A ring of structures, here called anchors, is invariably found underlying polyps (Fig. 1D–F). Anchors are circular in outline (12.0 +/− 2.4 µm^2^, n =  145 in area) and extend to a height of 3.5 +/− 0.7 µm (n = 207). In regions where the chloe encompasses a polyp as well as adjacent stolons, anchors are found both in the elliptical pattern found in the original polyp, and in a pattern ringing the perimeter of stolonal regions underlying the chloe (Fig. 1E, F). Anchors are attached to the substratum and are not continuous with the hydrorhizal walls. When living tissues are removed by treatment with KOH, the anchors remain. Anchors autofluoresce with the same intensity as perisarc in all three wavelength bands measured (Figure S1A–D). In KOH digested specimens, Calcafluor White (Figure S1E), Congo Red (Figure S1F), and wheat germ agglutinin (Fig. 1) were found to stain the anchors. Anchors were not found at the base of the youngest polyp buds.

#### Muscle fibers at the polyp stolon junction

**Figure 2:**
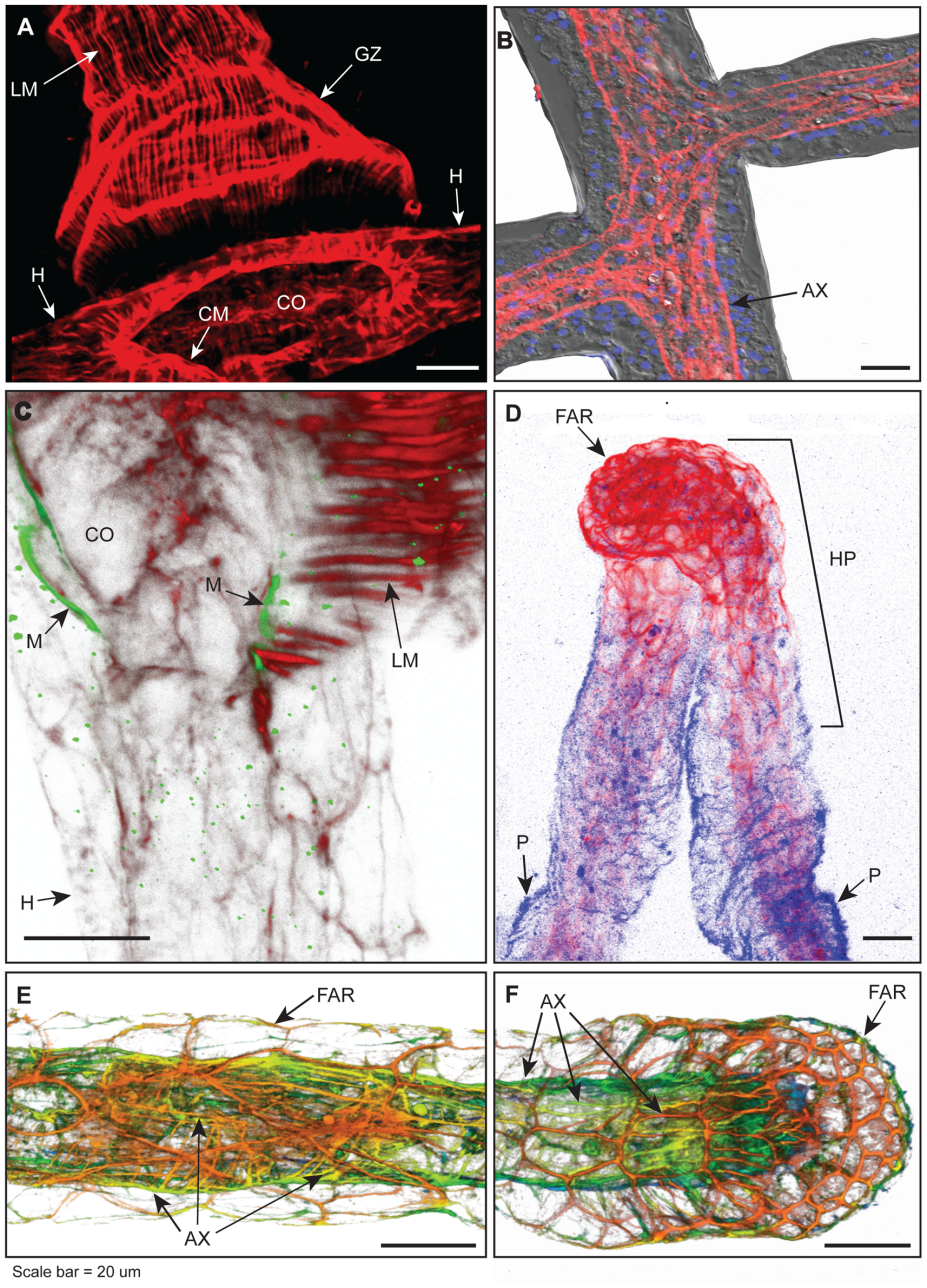
Stolons, hyperplastic stolons and the polyp-stolon junctions. (A) Projection of phalloidin stained gastrozooid, showing longitudinal muscle fibers and circular muscle fibers, the hydrorhiza from which the polyp emanates, and the location of the chloe. Note that polyp-stolon junction is a simple aperture, lacking specialized valves. (B) A portion of the hydrorhiza showing continuity of axial muscle fibers at two stolonal junctions. Differential interference contrast, grey, axial muscle fibers stained with phalloidin, red, and nuclei, DAPI stained in blue. (C) Projection of polyp stolon junction at the chloe showing longitudinal muscle strands of gastrozooid labeled with phalloidin, red, and mesoglea labeled with antibodies specific for *Hydra* collagen IV, green. Note that longitudinal muscle strands do not traverse mesoglea. (D) Projection of two hyperplastic stolons, labeled with phalloidin, red. The perisarc, blue, was visualized by autofluorescence. Note extensive meshwork of F-actin rings at tip. (E) Depth coded projection of phalloidin stained stolon at region proximal to the stolon tip, showing F-actin rings of the basal ectodermal cells where they adhere to the substratum (see also Figs. 4A,D) and gastrodermal axial muscle fibers. (F) Depth coded projection of phalloidin stained stolonal tip showing terminus of gastrodermal axial muscle strands and F-actin rings at stolonal tip. (E, F) Color code: red ca. 0–2 µm from substratum; orange 3–6 µm, yellow-green 7–10 µm, blue >10 µm. Optical depth, number of sections: (A) 27 µm, 14; (C) 8 µm, 10; (D) 40 µm, 10; (E) 17 µm, 43; (F) 18 µm, 25. (A–F) Scale: 20 ∶m. AX, axial muscle fibers; CO, opening of chloe; CM, circular muscle fibers; FAR, F-actin ring; GZ, gastrozooid; H, hydrorhiza; HP, hyperplastic stolons; LM, longitudinal muscle fibers, M, mesoglea, P, perisarc.

**Figure 3:**
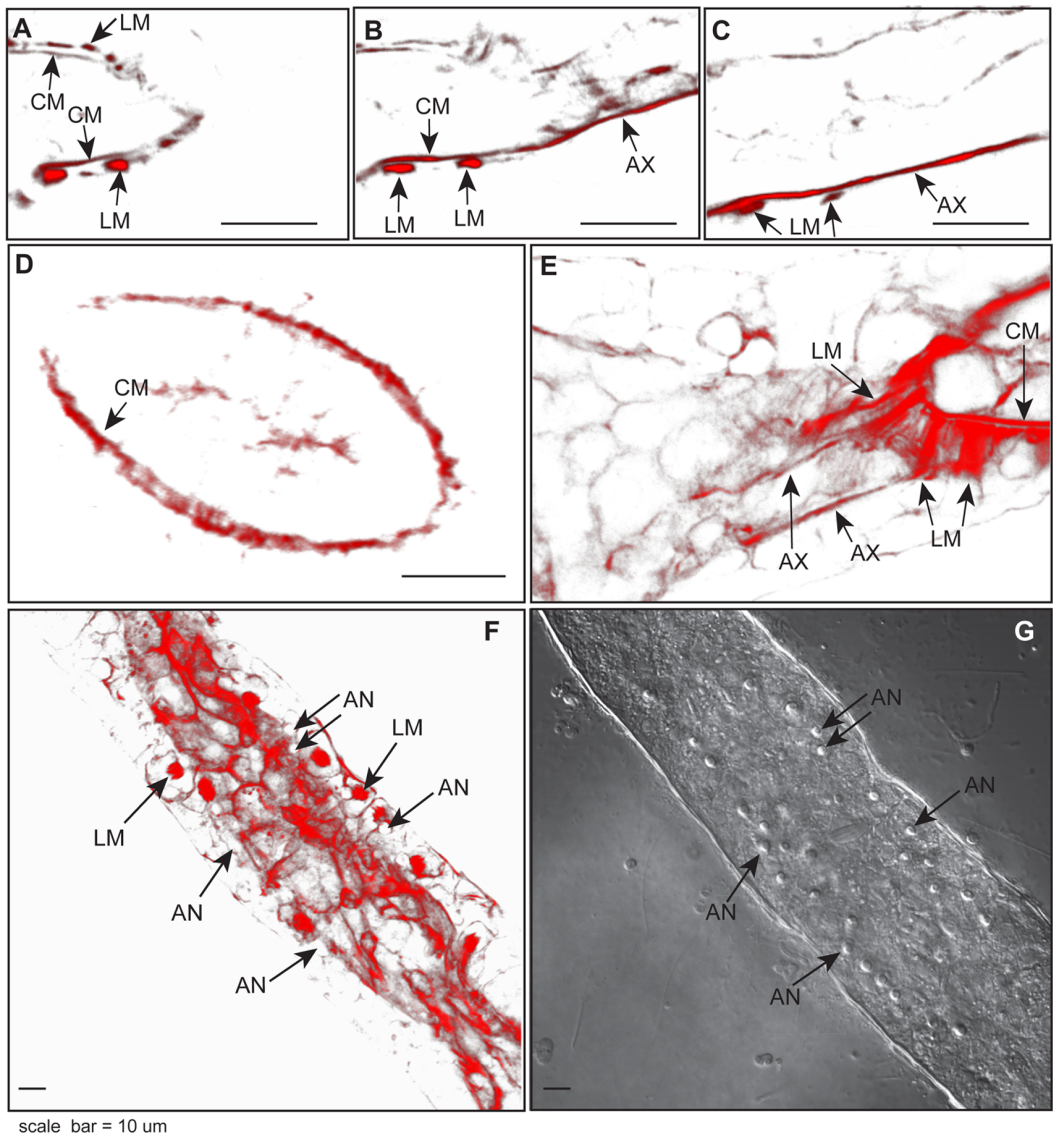
Polyp-stolon junction. (A–C) Phalloidin stain of the same stolon junction shown at (A) 20 µm, (B) 16 µm, (C) 8 µm above substratum, showing the disappearance of epidermal longitudinal muscle fibers of the polyp and the extension of gastrodermal circular muscle fibers of the polyp into the axial muscle strands of the stolon. (D) Optical section of a phalloidin stained polyp bud showing gastrodermal circular muscle fibers, but no longitudinal muscle fibers. (E) Projection of phalloidin stained muscle fibers at the base of a gastrozooid. Note that longitudinal muscle strands of the polyp project into broad fans at the junction with the proximal-distal axis of the stolon. (F) Projection of unusual phalloidin stained polyp-stolon junction showing longitudinal muscle fibers extending to the base of gastroderm. Position of anchors indicated. (G) Differential interference contrast (DIC) ιμαγε of the same stolon as (F) showing that the muscle strands labeled in (F) do not overly anchors. Optical depth, number of sections: (E) 1.3 µm, 3; (F) 2.7 µm, 3. Scale: 10 µm. AN, anchors; AX, axial muscle fibers; CM, circular muscle fibers; LM, longitudinal muscle fibers.

No specialized valves appear at the polyp-stolon junction (Fig. 2A). Optical sections just above the chloe reveal the circular muscle fibers of the polyp as the expected continuous ring internal to regularly spaced longitudinal muscle strands (Fig. 3A). At the level of the gastrovascular canal of the stolon, the circular muscle fibers of the polyps extend to run as axial strands along the proximal-distal axis of the stolon (Fig. 3B,C). In polyp buds, longitudinal fibers are absent, but circular muscle fibers are distinct and continuous with the axial fibers of the underlying stolon (Fig. 3D). Optical sections of the polyp-stolon junction of a mature gastrozooid and of a polyp bud are available as Movies S1 and S2, respectively.

The longitudinal muscle strands of the polyp broaden into fans at the chloe (Fig. 3E). A 3D rendering of optical sections of longitudinal muscle strands double stained for mesogleal collagen IV shows that the strands terminate in the mesoglea of the stolon (Fig. 2C). In the majority of cases longitudinal muscles strands can only be visualized in apical regions of the stolon. Infrequently muscle fibers were found to extend to the basal side of the stolonal gastroderm, but in no case did they extend through the epidermis to either the anchors or the substratum (Fig. 3F,G). The longitudinal muscle strands of the polyps have not been seen to extend axially along the length of the stolon.

**Figure 4:**
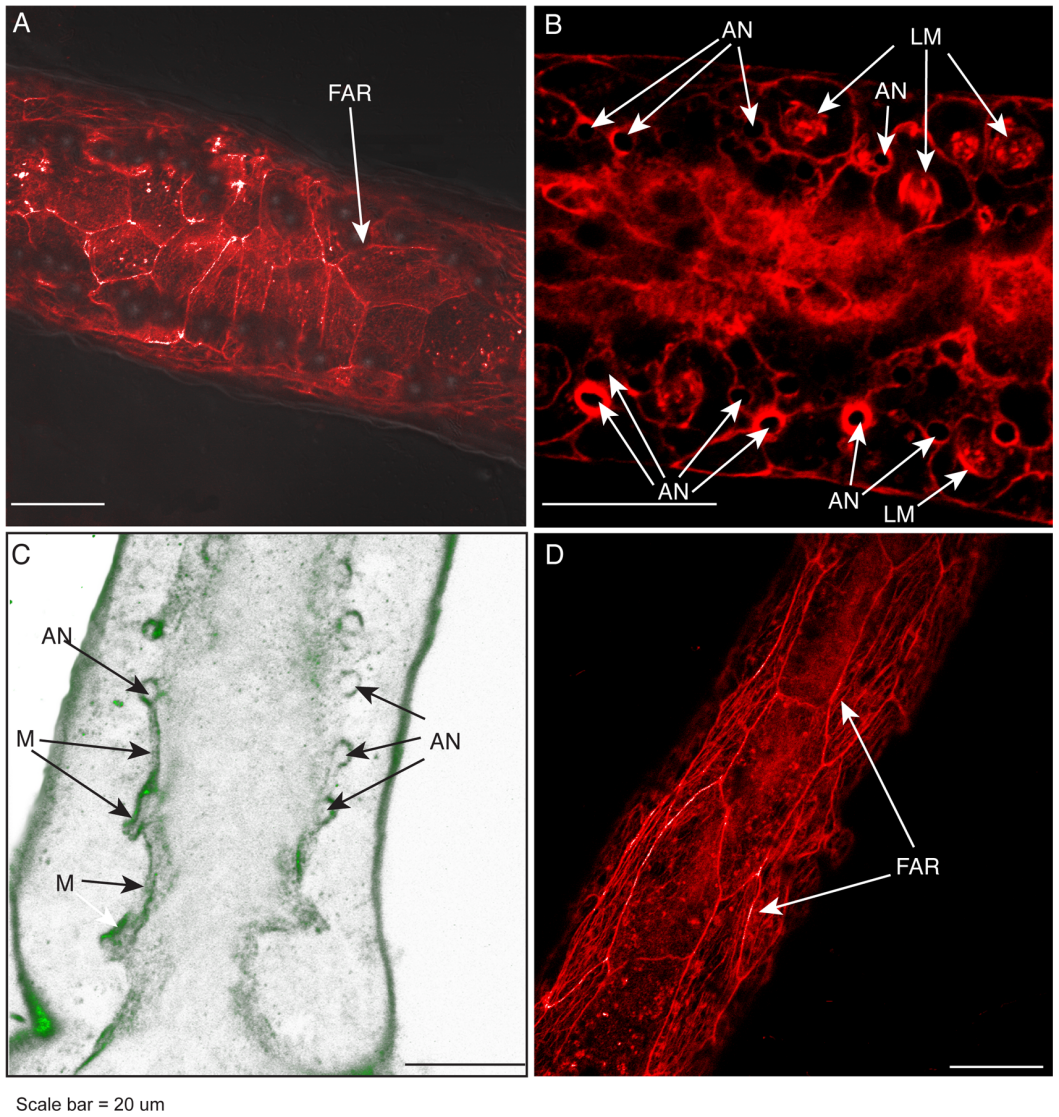
Basal epidermal actin, anchors, and mesoglea. (A) Phalloidin stained F-actin rings in the basal epidermis beneath a polyp-stolon junction. (B) Phalloidin stained F-actin fibers surrounding some, but not all, anchors. Diffuse staining in the center of stolon is gastrodermal. (C) Immunohistochemical staining of collagen IV showing mesoglea (M) overlying and surrounding anchors. Optical depth, number of sections: 3 µm, 4. (D) Phalloidin stained F-actin rings in basal epidermis of stolon at positions proximal to the stolon top. Scale: 20 µm. AN, anchors; FAR, F-actin ring; LM, longitudinal muscle strands; M, mesoglea.

The relationship of anchors to F-actin fibers and to the mesoglea was explored. In optical sections nearest the substratum, F-actin fibers are seen in polygonal pattern (Fig. 4A). F-actin cytoskeletal fibers also extend within basal epidermal cells where they are sometimes found to encircle the anchors (Fig. 4B, Movie S3). Optical sections stained for collagen IV indicate that mesoglea lies above the basal epidermal F-actin staining and that anchors are pegged to the mesoglea (Fig. 4C, Movie S4).

### The Stolon

F-actin staining of the stolon revealed extensive gastrodermal muscle strands (Fig. 2E), terminating at the stolonal tip (Fig. 2F). Thick fibers run along the proximal–distal axis of stolon radially or helically distributed around the stolonal lumen. Finer fibers crisscross the axial bands. Muscle fibers are continuous at stolonal branch points (Fig. 2B). In addition to the axial muscle fibers, F-actin staining was observed in a polygonal pattern in the regions of epidermal cells where the cells contact the substratum (Figs. 2E, 4A,D). This polygonal staining was found in all stolons at all locations in the colony.

### The Stolon Tip and Polyp Bud

**Figure 5:**
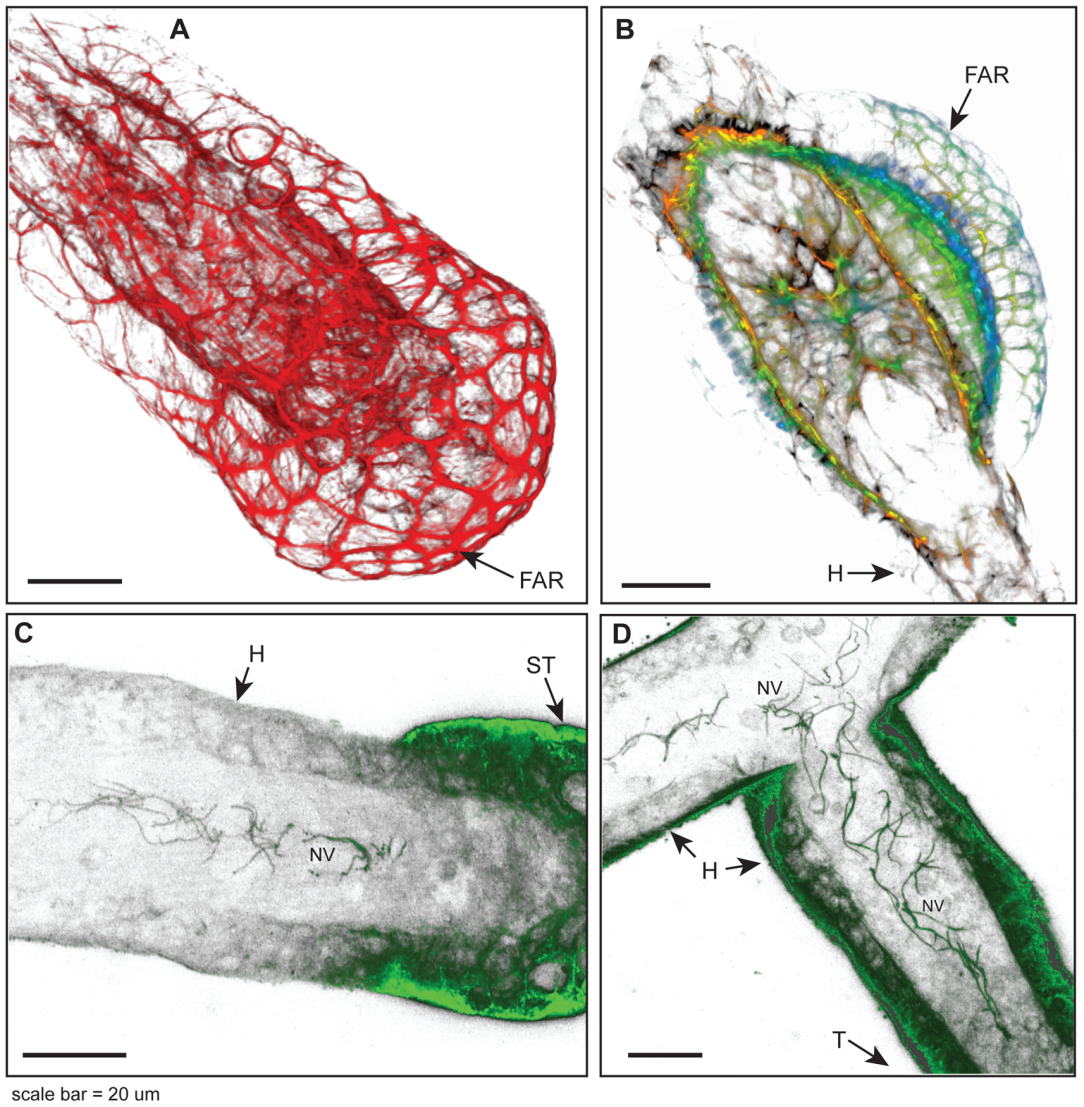
Stolon tip, polyp bud, and nerves. (A) Three-dimensional (3D) reconstruction of phalloidin stained stolonal tip showing F-actin ring structures in apical region of tip epidermal cells. (B) 3D reconstruction of polyp bud showing epidermal F-actin rings. Color code for panel B: red ca. 0–2 µm from substratum; orange 3–6 µm, yellow-green 7–10 µm, blue >10 µm. Projection of gastrodermal nerves, detected immunohistochemically, in locations (C) just proximal to the stolon tip and (D) extending into mother stolon from which a stolon tip had recently developed. Optical depth, number of sections: (A) 22 µm, 52; (B) 20 µm, 11; (C) 8 µm, 11; (D) 6 µm, 9. Scale: 20 µm. FAR, F-actin ring; H, hydrorhiza; NV, nerves; ST, stolon tip.

The apical surfaces of epidermal cells of both the stolon tip and the polyp bud were found to stain positively for F-actin. Three-dimensional rendering of optical sections revealed a distinctive F-actin meshwork at the tip and bud, comprised of polygons of interconnected actin fibers (Fig. 5A,B, Movies S5 and S6). This meshwork, which is not evident elsewhere, was found to cap the entire polyp bud and to surround the stolon tip from the apical upper epidermis to the lower basal epidermis. Hyperplastic stolons, those induced by allogeneic or xenogeneic encounters, bear a similar F-actin ring meshwork (Fig. 2D). The hyperplastic stolons differ from free growing tips in two respects. The perisarc does not extend to the hyperplastic stolonal tip and the distinctive cytoskeletal epidermal architecture extends proximally to a much larger extent.

### Nerves

Tubulin staining revealed the stolonal tip is enervated (Fig 5C,D). Nerves were found in the gastroderm in a region just proximal to the tip when the tip is an expansion phase (Fig. 5C) and abutting the tip at the depth of the retraction phase. Nerves are found to extend proximally for a variable distance (mean =  76.3 +/− 45.1 µm, n = 10) from the tip. In cases of new stolon branches, nerves have been observed to extend from the tip into the parental stolon (Fig. 5D). Nerves were not observed in other sections of the hydrorhiza under the conditions used to visualize nerves at the stolon tips.

## Discussion

### Anatomical Observations

Phalloidin staining of the hydrorhiza of *Podocoryna carnea* has revealed an extensive network of hydrorhizal muscular strands. With one exception [31], all earlier attempts have failed to detect muscle fibers in stolons [6], [15], [32]–[35]. The failure to detect muscle fibers, including in a species closely allied to the species we have observed [32], likely speaks to the far greater resolution provided by the use of confocal microscopy.

The stolonal musculature was found to be continuous with the circular muscle strands of the polyp and to extend in a series of gastrodermal axial fibers terminating in blind ends at stolonal tips. Our observation that the polyp bud has circular muscle fibers suggests that the circular muscle strands of newly budded polyps derive from the stolonal musculature. We were unable to unambiguously detect epidermal muscle fibers in the stolon.

We find that that the longitudinal muscle strands of the polyp terminate in the mesoglea of the underlying stolon. These muscle fibers widen at their base. If circular muscle strands were to contract at this location, this arrangement would serve to close off the gastrovascular cavity of the polyp from that of the stolon at the chloe. That such closure can be accomplished is known from the fact that rapid polyp contractions made by hydroids when perturbed are volume-preserving [3], [36]. Prior authors have assumed that the epidermal longitudinal muscle fibers of the polyps were pegged to the substratum via the anchors [37], presumably reasoning that the muscular contraction must be resisted in some fashion and the surface provides the most obvious solution. Our finding that the longitudinal fibers of the polyp terminate in the mesoglea would at first glance seem mechanically implausible. Yet stolons are fluid-filled and when the longitudinal muscle strands of the polyp are contracting, gastrovascular fluid is being ejected from the polyp into the stolon, so the stolon is fully expanded [38]. As water is essentially incompressible, a hydrostatic skeleton opposes the longitudinal muscle fibers.

Our observations indicate that the junction between the polyp and the stolon lacks valves. Rather the junction is simply a blind hole at the base of the polyp. Observations of polyps of different sizes show that the chloe is remodeled as stolons grow into proximity with the polyp. This remodeling requires that the apical perisarc of adjacent stolons is digested to provide a conduit from the stolon to the polyp, which accords with the finding in *Hydractinia* that the basal epidermis of polyps express chitinase [39].The appearance of additional anchors circumscribing regions of adjacent stolons that have come to empty into a single chloe indicates that the polyp base is also remodeled as the polyp enlarges.

The anchors observed at the polyp stolon junction have been observed by others [37], but ours is the first set of anatomical observations. The anchors are not living tissue, as indicated by their presence following KOH digestion. The fact the anchors autofluoresce with the same intensity as does perisarc and that, like perisarc, they stain positively for chitin using Calcofluor White, Congo Red, and wheat germ agglutinin suggest the anchors are localized perisarc secretions. Optical sectioning of a large number of polyps (n = 152) did not reveal a single instance of either the axial muscle strands of the stolonal gastroderm or the longitudinal muscle strands of the polyp attaching to the anchors. Indeed, the only F-actin staining found in association with the anchors were cytoskeletal fibers of the basal epidermis that were sometimes found encircling anchors. Our observations of collagen IV staining at and around the apical surface of the anchors indicate that these locations serve to peg the mesoglea to the surface.

The epidermal cells of the hydroid stolonal tip and polyp bud undergo regular oscillations in orientation and volume as the stolon or bud elongates [11]–[14]. The oscillations are a property of the tip and bud tissues, as isolated stolons or buds continue to display essentially similar movements [8]–[11], [40]. We have found that the tip and bud regions are characterized by an extensive intracellular meshwork of F-actin fibers that border and encase the apical margin of epidermal cells. The fact that the meshwork is bound into a coherent multicellular structure is suggestive of adherens junctions. Early electron micrographs indicate cytoplasmic extensions to junctions at the apical margin of stolon tips in *Cordylophora* (Figure 3 in Overton, 1963). Further support for this interpretation is found in the fact that cnidarian genomes include coding sequences for genes similar to the β-catenin, adherens junction protein p120, and cadherins necessary to support adherens junctional rings [41], [42].

### Functional Implications

Our anatomical observations bear on three distinct functional issues.

#### Tip and bud pulsations

The motive forces governing the regular oscillations in tip and bud cell orientation remain unknown. Beloussov and colleagues have showed that the apical surface of tip epidermal cells move as a coherent unit and that changes in cell orientation are largely determined by changes on the angle subtended between the mesoglea and the lateral margins of tip and bud epidermal cells [12]–[14]. Campbell [43], working with the holdfasts of *Corymorpha*, first noted that tip locomotion requires that apical surfaces of the tip cells have a resisting surface and suggested that the perisarc, secreted at the tip, served this purpose. Yet perisarc at the growing tip is not yet hardened and we suggest that the adherens junctional ring may serve this role. The interpretation is supported by the observation that hyperplastic stolons possess the extensive F-actin meshwork, yet appear to secrete minimal perisarc at their tips. Moreover, in athecate hydroids such as the *Podocoryna carnea* colonies we study here, polyps are free of perisarc, and yet polyp buds undergo pulsations.

Early studies showed that cytochalasin B eliminates tip pulsations [10], suggesting a role for F-actin mediated movement. Adherens junction rings are known to be contractile in a number of systems [44]–[47]. If the tip and bud adherens junctional rings are contractile they may provide the motive force responsible for pulsations. This suggestion can be directly tested by treating growing tips and polyp buds with the myosin II specific inhibitor blebbostatin.

#### Lumen closure

The stolonal lumen changes in radius as fluids are pumped into and out of polyps [2]–[5]. In the youngest peripheral stolons, the lumen may close completely while in older, more central parts of a colony the lumen oscillations are of smaller amplitude. We are aware of only one attempt to explain how the lumen closes. Schierwater, et al [32] observed that the apical surface of gastrodermal digestive cells are densely populated by structures they called ‘zipper-like organelles’ (ZLO). They proposed that ZLO's acted as contractile vacuoles that open to admit gastrovascular fluid, thereby expanding the volume of gastrodermal cells and closing the lumen [32]. However, the ZLO's of Schierwater et al [32] are identical in ultrastructural detail with that of discoidal coated vesicles, a feature previously described in hydroid digestive cells [48]. Discoidal coated vesicles act to increase the surface area for absorption. When these vesicles are internalized within the cell they do so without an accompanying change in cell volume beyond that of the nutrients adhering to the coated vesicle surface [48]. Another fact mitigating against the interpretation of Schierwater, et al [32] is the simple observation that fluid is readily observed moving axially as the stolon lumen closes. If the fluid were absorbed into the gastrodermal cells, lumens would not flush axially.

The opening and closing of the lumen need not be an active process. The gastroderm and mesoglea could be behaving as passive elastic materials, expanding when lumen pressure, generated by polyp pumping, exceeds the some resting radius and closing to this resting radius when lumen pressure declines. However, anecdotal observations have been made of stolon branches with closed lumens communicating directly with parent stolons within which gastrovascular fluid is actively flowing. This can only be accounted for if some active process is keeping the stolon branch closed.

Our observations suggest such an active mechanism for lumen closure. If the axial gastrodermal muscle fibers were to contract, gastrodermal cells would be expected to change shape from rhomboidal to columnar with the consequence that the lumen itself would close to the extent that hydroplasmic pressures in the lumen permitted. When a stolon is filling, one or more polyps are pumping fluid into the hydrorhizal network. The pumping of polyps requires the contraction of longitudinal muscle strands and presumably relaxation of some circular muscle fibers. If the state of the circular muscle fibers at the base of a pumping polyp were transmitted to the adjacent stolon, then the axial muscle strands of the stolon would be expected to relax as the polyp empties and the stolon fills. When gastrovascular flow reaches the tip, further expansion of the stolon is no longer possible and the axial fibers of the stolon contract, returning fluid to the polyp in response to which longitudinal muscle strands of the polyp relax to accommodate the incoming fluid. Thus the pattern of polyp driven volume oscillations in hydroplasmic flows is consistent with the suggestion that lumen closure involves muscular contraction. Finally, if muscular contraction is responsible for the shape changes in gastrodermal cells, the entire gastrodermal epithelia must shorten when the stolon lumen empties. Studies of tip pulsations show just such a retraction phase, with the maximal extent of retraction occurring when the stolon lumen has emptied [16].

Our results have localized nerves to stolonal tips. Severing of a tip results in the immediate closure of the stolonal lumen and its disuse until the tip is fully reconstituted and active (unpublished observations). If adherens junctional ring contractions do drive tip pulsations, the nerves are not likely involved in these tip movements, since contraction of adherens junctional rings is driven by Ca^2+^–calmodulin dependent myosin light chain kinase phosphorylation of myosin II [49]–[52]. Rather, we suggest that the nerves found at stolon tips trigger the stolon axial muscle fiber contraction, initiating return flow to polyps.

An additional role of stolonal muscle fibers may be to increase the rigidity of the mesoglea in the axial direction and, hence reduce any tendency of the stolon lumen to flutter or locally collapse as the water returns from the stolons to the polyps.

### Developmental origin of the basal epidermis and of the anchors

The developmental origin of the basal epidermis of the hydroid hydrorhiza seems not to have been previously addressed. The hydrorhiza is anchored to the substratum by the differentiation of desmocytes which secrete rivets that attach the underlying surface to the overlying mesoglea, after which the desmocyte dies and the rivet lies between adjacent basal epidermal cells [1], [53], [54]. Rivets form on the basal epidermis in regions proximal to the tip. This anchoring arrangement would seem to preclude mitotic activity in the basal epidermis in the immediate regions proximal to the tip. Cell marking studies, conducted on the time scale of seconds up to 20 hours, show that epidermal cells at the tip remain stationary, while apical and lateral cells proximal to the tip region but distal to the youngest polyp move toward the tip [9], [11], [55]. If the basal epidermis is not growing by mitosis in regions proximal to the tip and if epidermal cells on the flanks and apical surfaces of the stolon are moving distalwards, a plausible origin of the basal epidermis is that upper and lateral epidermis is transformed, over a time scale of days, into basal epidermis with the same motion as a track on a treadmill. This model is reminiscent of the well-known tissue dynamics of the polyp epidermis. The polyp is comprised of a two radially symmetric, continuously moving and differentiating cell sheets. Classical studies in *Hydra* have shown that mitosis is concentrated at mid-gastric regions and new cells move toward the hypostome and tentacles above this zone and towards the foot below it, with cells being shed at the termini [56]–[58]. Since stolon tips have not been observed to lose cells, we suggest that epidermal cells on stolon flanks and apical regions become basal epidermal cells as they round the tip. Our suggestion that the basal epidermis is derived from apical and lateral epidermis is further supported by the observation that the substratum-facing surface of basal epidermal cells displays the same putative adherens junctional rings characteristic of the tip. The ‘treadmill’ hypothesis can be directly tested by marking cells and following their fate over the time course of days.

The anchors observed here suggest another role for the basal stolon ectoderm. We speculate that anchors arise as do rivets [1], [53], [54]. Rivets are secretions of specialized basal ectodermal cells called desmocytes, which, like anchors, serve to link the mesoglea to the substratum. Any correspondence likely ends there, as anchors are peridermal secretions and rivets are not. Our observation that the youngest polyp buds lack both anchors and longitudinal muscles tempt us to suggest the possibility that longitudinal muscle maturation may generate mechanical forces that induce the basal ectodermal epithelium to secrete anchors.

## Supporting Information

Figure S1 **Anchors (arrows).** (A–D) Same stolon visualized in (A) DIC and (B–D) autofluorescence at different wavelengths. (B) Ex., 488 nm, Em. 500–550 nm, (C) Ex., 760–780 in 2-photon configuration, chameleon IR laser, no pinhole, Em. 435–485, (D) Ex., 561 nm, Em. 575 nm. KOH-digested perisarc showing anchors stained with (E) Calcofluor White and (F) Congo Red. Scale: 10 µm.
(TIF)

Movie S1 **Polyp-stolon junction.** Movie comprised of serial optical sections of a phallodin stained F-actin (red) and DAPI stained nuclei (blue) through a polyp stolon junction. Early frames are apical. Note the disappearance of longitudinal muscle strands of the polyp and the origin of the axial muscle strands of the stolon from the circular muscle strands of the polyp. Optical depth, number of sections: 6 µm, 12. AX, axial muscle fibers; LM, longitudinal muscle fibers; CM, circular muscle fibers.
(MOV)

Movie S2 **Polyp bud.** Movie comprised of serial optical sections of a phallodin stained F-actin (red) and DAPI stained nuclei (blue) through a polyp bud. Early frames are basal. Note the absence of longitudinal muscle strands of the polyp. Optical depth, number of sections: 22 µm, 10. AX, axial muscle fibers; CM, circular muscle fibers.
(MOV)

Movie S3 **Basal epidermis and anchors.** Movie comprised of serial optical sections of phallodin stained F-actin (red) in basal epidermis. Early frames are basal. Note that some anchors are surrounded by F-actin fibers and that these all lie beneath the stolonal lumen. Optical depth, number of sections: 7 µm, 18. AN, anchors; AX, axial muscle fibers; SL, stolonal lumen.
(MOV)

Movie S4 **Mesoglea and anchors.** Movie comprised of serial optical sections of immunohistochemically stained collagen IV (green). Optical section overlay DIC images showing anchors. Note the mesoglea was found draped over and around anchors. Optical depth, number of sections: 18 µm, 24. AN, anchors; M, mesoglea; SL, stolonal lumen.
(MOV)

Movie S5 **Stolon tip.** Movie comprised of serial optical sections of phallodin stained F-actin (red) in a stolon tip. Early frames are basal. Optical sections terminate at the upper surface of the gastrovascular lumen and so do not include the upper epidermal surface. Note the distinctive F-actin ring meshwork surrounding the apical margins of tip epidermal cells. Optical depth, number of sections: 21 µm, 52. FAR, F-actin rings; SL, stolonal lumen.
(MOV)

Movie S6 **Polyp bud.** Movie comprised of serial optical sections of phallodin stained F-actin (red) in a polyp bud. Bud has just begun to produce longitudinal muscles, hence is somewhat more advanced in development than the bud shown in Figure 3D. Early frames are basal. Note the distinctive F-actin ring meshwork surrounding the apical margins of bud epidermal cells. Optical depth, number of sections: 19 µm, 11. FAR, F-actin rings; CM, circular muscle fibers; LM, longitudinal muscle fibers.
(MOV)
