# Supplementary figures and images for: Muscular Anatomy of the Podocoryna carnea Hydrorhiza
Source: PLoS One. 2013 Aug 14;8(8):e72221. doi: 10.1371/journal.pone.0072221 (PMC3743812; doi:10.1371/journal.pone.0072221)

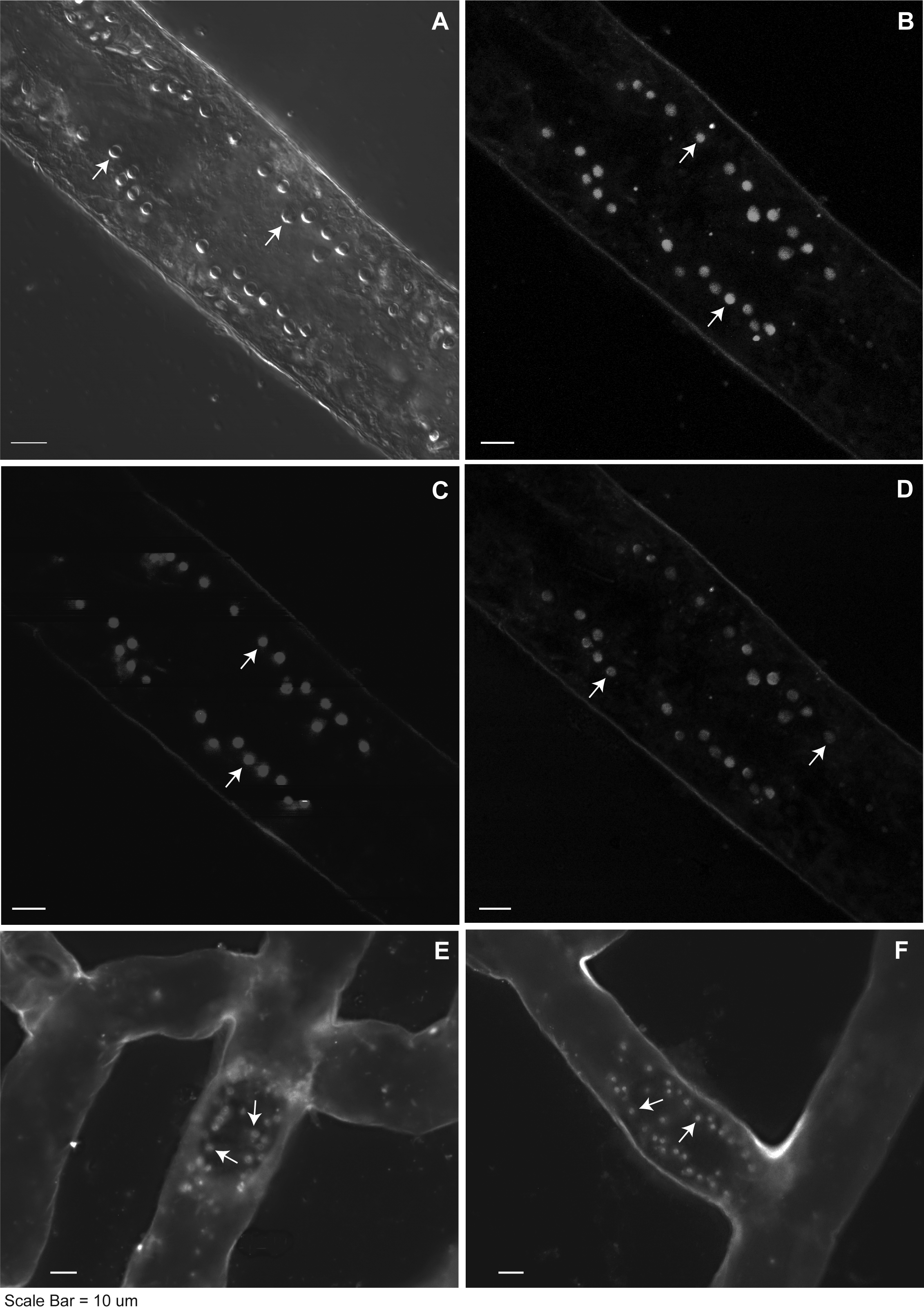

Supplement: Figure S1 — Anchors (arrows). (A–D) Same stolon visualized in (A) DIC and (B–D) autofluorescence at different wavelengths. (B) Ex., 488 nm, Em. 500–550 nm, (C) Ex., 760–780 in 2-photon configuration, chameleon IR laser, no pinhole, Em. 435–485, (D) Ex., 561 nm, Em. 575 nm. KOH-digested perisarc showing anchors stained with (E) Calcofluor White and (F) Congo Red. Scale: 10 µm. (TIF) [file pone.0072221.s001.tif]
